# Development of domperidone bilayered matrix type transdermal patches: physicochemical, in vitro and ex vivo characterization

**Published:** 2010

**Authors:** S.K. Madishetti, C.R. Palem, R. Gannu, R.P. Thatipamula, P.K. Panakanti, M.R. Yamsani

**Affiliations:** Centre for Biopharmaceutics and Pharmacokinetics, University College of Pharmaceutical Sciences, Kakatiya University, Warangal- 506009 (A.P.), India

**Keywords:** Domperidone, Bilayered Transdermal patches, In vitro release, Ex vivo permeation

## Abstract

**Background and the purpose of the study:**

Domperidone (DOM) is a dopamine- receptor (D_2_) antagonist, which is widely used in the treatment of motion-sickness. The pharmacokinetic parameters make DOM a suitable candidate for transdermal delivery. The purpose of the present investigation was to develop transdermal delivery systems for DOM and to evaluate their physicochemical characteristics, in vitro release an ex vivo permeation through rat abdominal skin and their mechanical properties.

**Methods:**

Bilayered matrix type transdermal drug delivery systems (TDDS) of DOM were prepared by film casting technique using hydroxypropyl methyl cellulose as primary and Eudragit RL 100 as secondary layers. Brij-35 was incorporated as a solubilizer, d-limonene and propylene glycol were employed as permeation enhancer and plasticizer respectively. The prepared TDDS were extensively evaluated for in vitro release, moisture absorption, moisture content, water vapor transmission, ex vivo permeation through rat abdominal skin, mechanical properties and stability studies. The physicochemical interaction between DOM and polymers were investigated by Differential Scanning Calorimetry (DSC) and Fourier Transform Infrared Spectroscopy (FTIR).

**Results:**

All the formulations exhibited satisfactory physicochemical and mechanical characteristics. The optimized formulation F6 showed maximum cumulative percentage of drug release (90.7%), permeation (6806.64 µg) in 24 hrs, flux (86.02 µg /hr/cm^2^) and permeation coefficient of 0.86x10^−2^ cm/hr. Values of tensile strength (4.34 kg/mm^2^) and elastic modulus (5.89 kg/cm^2^) revealed that formulation F6 was strong but not brittle. DSC and FTIR studies showed no evidence of interaction between the drug and polymers. A shelf life of 2 years is predicted for the TDDS.

**Conclusions:**

Domperidone bilayered matrix type transdermal therapeutic systems could be prepared with the required flux and suitable mechanical properties.

## INTRODUCTION

The transdermal route of administration has been recognized as one of the potential routes for local and systemic delivery of drugs. This route offers many advantages over the oral dosage form, such as improving patient compliance in long-term therapy, bypassing first-pass metabolism, sustaining drug delivery, maintaining a constant and prolonged drug level in plasma, minimizing inter- and intra patient variability, and making it possible to interrupt or terminate treatment when necessary (1). However, the highly organized structure of the stratum corneum forms an effective barrier to drug permeation, which must be modified if poorly penetrating drugs are to be administered. The use of chemical penetration enhancers significantly increases the number of drug molecules suitable for transdermal delivery (2, 3). In addition, the transdermal patch dosage form is user-friendly, convenient and painless, and generally leads to improved patient compliance. Intensive research has shown that transdermal route is a potential mode of delivery for lipophilic drugs in systemic circulation (4).

Domperidone is a dopamine- receptor (D) antagonist, widely used in the treatment of motion-sickness. In humans, peak plasma levels of domperidone occur within 10 to 30 min following intra-muscular injection and 30 min after oral (fasted) administration. It has been reported that it is rapidly absorbed after oral administration, but undergoes extensive first pass metabolism; leading to poor bioavailability of 15% (5). From both, physicochemical (low molecular weight 425.9g/mol, low dose 10 mg) and pharmacokinetic (absolute bioavailability about 10–20% and log P, 3.11) perspective, DOM was considered to be a suitable candidate for transdermal delivery.

In spite of several advantages offered by transdermal route, only a few drug molecules are administered transdermally because of the formidable barrier nature of stratum corneum (6). Two major approaches to increase transdermal permeation rate include physical techniques (iontophoresis, electroporation, sonophoresis, and microneedles) and use of chemical penetration enhancers such as solvents, surfactants, fatty acids and terpenes (7–10). Terpenes present in naturally occurring volatile oils appear to be clinically acceptable enhancers (11). Moreover, a wide variety of terpenes have been shown to increase the percutaneous absorption of a number of drugs (12). In the present study d- limonene was used as penetration enhancer, as reported earlier for some other drugs (13, 14).

In the initial trials which were made with monolayer patches, drug diffusion from the monolayer patches was observed. In order to prevent the drug diffusing from the surface of the patch, bilayered transdermal patches were developed. The objective of present investigation was development of bilayered transdermal therapeutic systems for DOM and to evaluate physicochemical, mechanical properties, in vitro release and ex vivo permeation through rat abdominal skin

## MATERIAL AND METHODS

### 

#### Materials

Domperidone, Hydroxypropyl methylcellulose (HPMC E15) and Eudragit RL 100 (ERL 100) were gift samples from Torrent pharmaceuticals (Baroda, India) and Dr. Reddy's laboratories (Hyderabad, India) respectively. Transcutol and d-limonene were purchased from Gattefosse, (France) and Hi Media (Mumbai, India) respectively. All other chemicals were of analytical grade.

#### Preparation of rat abdominal skin

Albino rats weighing 150–200 g were sacrificed using anesthetic ether. Heat separation technique was used to prepare the epidermis (15), which involved soaking the entire abdominal skin in water at 60 oC for 45 sec, followed by careful removal of the epidermis. The epidermis was washed with water and used for ex vivo permeability studies.

#### Effect of d-limonene on permeation of DOM

Effect of d-limonene on permeation of DOM through rat abdominal skin was studied using franz diffusion cell. DOM solution [5 mg in 4 ml of the phosphate buffered saline (PBS) of pH 5.6 containing polyethylene glycol (PEG 400)] was placed in the donor compartment containing different concentrations of d-limonene (0, 4, 8, 12 and 16% v/v). The 0% d-limonene served as control and PEG 400 was used to solubilize DOM. The receiver compartment contained 25 ml of 40% v/v PEG 400 in PBS of pH 7.4 and the contents were stirred at 500 rpm using magnetic stirrer. The entire assembly was kept at37±0.5 oC. Samples of 1 ml were collected at preset time points upto 24 hrs and replenished with fresh buffer. The samples were filtered through 0.45 µ syringe filter (Sartorius AG, Goettingen, Germany) and drug content in the samples was determined by high performance liquid chromatography (HPLC) (16).

#### HPLC methodology for DOM

HPLC determination of DOM was performed using Shimadzu LC 20AT solvent delivery pump equipped with a 20 µl loop, rheodyne sample injector and UV- visible detector. Samples were chromatographed on a reverse phase C_18_ column (250×4.25 mm, 5 µm particle Phenomenex, Gemini column). Elution was conducted with a mobile phase of acetonitrile: water at ratio of 31:69 v/v containing 0.25% v/v or of triethyl-amine of pH 2.5 at a flow rate of 1 ml /min. A calibration curve was plotted for DOM in the concentration range of 0.5–10 µg ml^−1^. A good linear relationship was observed between the concentration of DOM and the peak area of DOM (r^2^=0.999). The required studies were carried out to estimate the precision and accuracy of the HPLC method.

#### Development of bilayered transdermal systems

Bilayered matrix type transdermal patches were prepared using solvent casting technique (17) with HPMC E15 as primary polymeric layer, Eudragit RLPO as secondary polymeric layer, Brij-35 (a non-ionic surfactant) as a solubilizer and propylene glycol as plasticizer. Primary polymer was added to 20 ml of the solvent mixture (dichloromethane and methanol, 1:1) and allowed to stand for 6 hrs to swell. Brij-35 and DOM were dissolved in 5 ml of solvent mixture and added to the polymeric solution. Measured quantity of d-limonene (12% v/v) was added as penetration enhancer. This was set aside for 2 hrs to remove entrapped air, then transferred to a petri plate, and dried at room temperature. The secondary polymeric solution was prepared by dissolving 300 mg of Eudragit RLPO and 60 µl of propylene glycol in 15 ml of solvent mixture and poured on the primary polymer layer and allowed to dry at room temperature. The developed patches were removed carefully, cut to size (each having an area of 3.14 cm^2^), and stored in a desiccator. The composition of the patches is shown in table 1. Patches were subjected to weight, thickness variation and content uniformity.

**Table 1:** Composition of Domperidone bilayered transdermal patches.

Component	F1	F2	F3	F4	F5	F6	F7	F8	F9
**Primary Layer**									
Domperidone (mg)	250	250	250	250	250	250	250	250	250
HPMC E15 (mg)	2000	2000	2000	2500	2500	2500	3000	3000	3000
Brij-35 (µL)	60	120	180	60	120	180	60	120	180
d-limonene (µL)	240	240	240	300	300	300	360	360	360
Propylene glycol (µL)	300	300	300	375	375	375	450	450	450
**Secondary Layer**									
Eudragit RLPO (mg)	300	300	300	300	300	300	300	300	300
Propylene glycol (µL)	60	60	60	60	60	60	60	60	60

#### Evaluation of physicochemical properties

Six films from each series were weighed individually and the average weight was calculated. The thickness of the patch was measured at six different points of the patch using digital gauze (Mitutoyo, Japan). Patches from each series of formulations (n=3) of 3.14 cm^2^ area were cut into pieces and weighed. The pieces were taken into a 100 ml volumetric flask, allowed to dissolve in 2 ml of dimethyl formamide and adjusted to 100 ml with 0.1N hydrochloric acid solution. The solution was filtered through 0.45 µm membrane filters and the drug content was analyzed using UV-visible spectrophotometer at 284 nm.

#### Moisture absorption study

The films were weighed accurately and placed in a desiccator containing 100 ml of saturated solution of aluminum chloride (79.5% RH). After 3 days, the films were taken out and weighed. The percentage of moisture uptake was calculated as the difference between the final and initial weight with respect to the initial weight (18).

#### Moisture content

The patches were weighed and kept in a desiccator containing calcium chloride at 40 ^o^C for 24 hrs. The final weight was noted when there was no further change in the weight of patch. The percentage of moisture content was calculated as a difference between initial and final weight with respect to the final weight (19).

#### Water Vapor Transmission Rate (WVTR) Studies

WVTR studies were performed according to the modified method described by Kusum *et al*. (18). Glass vials of equal diameter which were used as transmission cells were washed thoroughly and dried in oven. About 1 g of anhydrous calcium chloride was placed in the cells and the respective polymer film was fixed over the brim. The cells were accurately weighed and kept in a closed desiccator containing saturated solution of potassium chloride to maintain a relative humidity of 84%. The cells were taken out and weighed after 24 hrs. The amount of water vapor transmitted was determined using following formula:

1 $$\text{WVTR}=\frac{\text{Final weight}-\text{Initial weight}}{\text{Time}\times \text{Arera}}$$

Water vapor transmission rate is expressed as the number of grams of moisture gained/hr/cm^2^.

#### Measurement of mechanical properties

The film's mechanical properties were evaluated using a microprocessor-based advanced force gauge (Ultra Test, Mecmesin, UK) equipped with a 25 kg load cell. Film strips with dimensions of 60×10 mm and free from physical imperfections were held between two clamps positioned at a distance of 3 cm. During measurement, the top clamp pulled the strips at a rate of 2 mm/s until the film broke. The force required to break a film and elongation at a break were measured using dataplot software. The mechanical properties were calculated according to the following formulae (20).

2 $$\text{TS}(\text{K}{\text{g}}^{-\text{2}})=\frac{\text{Force at break}(\text{Kg})}{\text{Initial cross sectional area of the sample}(\text{m}{\text{m}}^{\text{2}})}$$

3 $$\frac{\text{E}}{\text{B}}(\%\text{m}{\text{m}}^{-\text{2}})\frac{\text{Increase in length}(\text{mm})}{\text{Original length}(\text{mm})\times \text{Cross sectional area}(\text{m}{\text{m}}^{\text{2}})}\times \text{100}$$

4 $$\text{EM}(\text{Kgm}{\text{m}}^{-\text{2}})=\frac{\text{Force at correspovding strain}(\text{Kg})}{\text{cross sectional area}(\text{m}{\text{m}}^{\text{2}})}\times \frac{\text{1}}{\text{correspovding strain}}$$

5 $$\text{Strain}=\frac{\text{Tensile strength}}{\text{Elastic modulus}}$$

where, TS, E/B and EM represents tensile strength, elongation at break and elastic modulus respectively.

#### In vitro drug release studies

**Figure 1:**
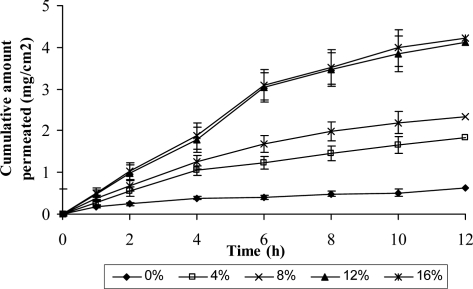
Effect of d-limonene concentration on cumulative permeation of DOM, Mean±S.D (n=3).

Franz diffusion cell with a surface area of 3.46 cm^2^ was used for in vitro release studies (21, 22). The transdermal patch was kept in the donor compartment and it was separated from the receptor compartment by dialysis membrane (Hi media M.W. cut off 5000). The donor and receptor compartment held together using clamp. The receiver compartment contained 25 ml of PBS of pH 5.6 containing 20% v/v of PEG-400, stirred at 500 rpm and temperature was maintained at 37±0.5 ^o^C. Samples of 1 ml were withdrawn at pre-determined time intervals and replenished with an equal volume of fresh medium. The drug content in the samples was determined by UV/ visible spectrophotometer at 284 nm. Cumulative percentage of the drug released were calculated and plotted against time (Figure 1).

#### Ex vivo permeation studies

Franz diffusion cell was used for ex vivo permeation studies and the skin was mounted between the two compartments of the diffusion cell with stratum corneum facing the donor compartment. The stratum corneum side of the skin was kept in intimate contact with the release surface of the TDDS under test. A dialysis membrane (Hi Media, M.W. cutoff 5000) was placed over the patch, in order to secure the it tightly in the way that will not get dislodged from the skin. The receiver phase contained 12 ml PBS of pH 7.4 containing 20% v/v PEG 400 whichwas stirred at 500 rpm on a magnetic stirrer and the whole assembly was kept at 37±0.5 ^o^C. Samples of 1 ml were withdrawn at pre-determined time intervals upto 24 hrs, the volume was replenished with an equal volume of fresh medium and analyzed by HPLC. Cumulative amounts of drug permeated in µg/cm2 were plotted against time and drug flux ( µg/cm^2^/hr) at steady state was calculated by dividing the slope of the linear portion of the curve by the area of the exposed skin surface (3.14 cm^2^) (22) and the permeability coefficient was deduced by dividing the flux by initial drug load. The target flux was calculated using the following equation (20).

6 $${\text{J}}_{\text{Target}}=\frac{\text{CssC}{\text{l}}_{\text{t}}\text{BW}}{\text{A}}$$

A, represents the surface area of the transderm(6a)l patch (i.e. 3.14 cm^2^); BW, the standard human body weight of 60 kg; C_ss_, the domperidone concentration at the therapeutic level (11.35 µg/l) (23) and the Cl_t_, the total body clearance (7–10 ml/min/kg); the calculated target flux value for DOM was 88.28 µg/cm^2^/hr.

#### Drug -polymer interaction study

In order to determine a possible interaction between DOM and the polymeric materials of the patches, infrared (IR) spectroscopy and differential scanning calorimetry (DSC) studies were carried out on pure substances and their physical mixtures. The IR spectra were recorded using an IR-Spectrophotometer (PerkinElmer FT-IR, PerkinElmer, Waltham, MA) utilizing the KBr pellet method. DSC studies were conducted using a Differential Scanning Calorimeter (Mettler-Toledo, Viroflay, France). The samples were scanned at 10 °C/min over the temperature range of 120 – 300 °C.

#### Stability studies

The stability studies were conducted according to the International Conference on Harmonization (ICH) guidelines (24). The optimized formulation F6 was wrapped in an aluminum foil and placed in stability chamber (Labtop, India) at a temperature of 40±0.5 ^o^C and 75±5% RH for 6 months. Samples were withdrawn at intervals of 1, 2, 3 and 6 months and analyzed for drug content and in vitro release.

## RESULTS AND DISCUSSION

### 

#### Effect of d-limonene on permeation of DOM

The effect of concentration of d-limonene on cumulative permeation of DOM through rat skin is shown in figure 1. Solution containing 12 and 16% v/v of d-limonene showed similar flux values (125.9±2.88 and 126.1±2.38 µg/cm^2^/hr) and permeability coefficients (25.1±0.12 and 25.2±0.17 cm hr^−1^×10^−2^). The flux values which were obtained with 8 and 12% v/v of d-limonene were significantly different (p<0.05) to lowest values and were obtained with 4 and 8% d-limonene (42.4±1.47 and 62.7±1.47 µg/cm2/hr) and control (10.8±1.06 µg/cm2/hr). The permeability coefficients obtained with 12 and 16% d-limonene were 12.4 and 12.5 times higher than that observed with control. The permeation of DOM was not affected by increasing d-limonene concentration from 12 to 16% v/v; hence in the preparation of patches, d-limonene was used at a concentration of 12% v/v.

#### Weight, thickness variation and drug content

The physicochemical properties (weight, thickness variation, drug content and water vapor transmission) of the transdermal patches are shown in table 2. The weight range of the patches were from 150.0±7.77 to 158.5±7.07 mg and the thickness ranges were 186.5±4.94 to 207.5±8.48µm. The results showed that the patches were uniform, as it was evidenced by RSD value, which were less than 6. The drug content ranged from 98.5±0.16 to 101.8±0.26%. All formulations were acceptable with regard to domperidone content.

**Table 2:** Weight, Thickness, Drug content and Water Vapor Transmission Values of DOM transdermal patches.

S.No	Formulation Code	weight^a^ (mg)	Thickness^a^ (μm)	Drug content^b^ (%)	WVT^b^ (g/cm^2^)×10^−3^
1	F1	150.0±7.77	197.5±3.53	100.8±0.17	3.81±0.05
2	F2	156.5±6.36	196.6±7.07	99.90±0.11	5.27±0.02
3	F3	153.5±3.53	186.5±4.94	101.5±0.16	5.31±0.08
4	F4	155.0±5.65	207.0±10.6	100.3±0.11	5.52±0.02
5	F5	150.8±2.82	203.5 ±11.2	101.8±0.26	5.11±0.06
6	F6	157.5±3.53	196.5±4.94	99.30±0.14	3.42±0.09
7	F7	152.5±2.12	202.0±4.24	101.1±0.21	3.87±0.06
8	F8	153.5±2.12	207.5±8.48	98.80±0.14	4.40±0.06
9	F9	158.5±7.07	195.0±7.77	98.51±0.16	6.81±0.07

a Results are mean±SD (n=6)

b Results are mean±SD (n=3)

#### Moisture content and moisture absorption studies

**Figure 2:**
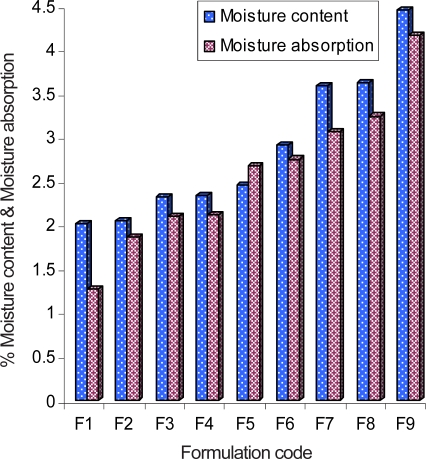
Moisture content and moisture absorption of domperidone bilayered transdermal patches.

The results for moisture content and moisture absorption studies are shown in figure 2. The moisture content in the patches ranged from 2.02±0.98% (F1) to 4.45±0.76 (F9).The moisture content in the formulations was found to be increased by increase in the concentration of HPMC. The moisture absorption in the formulations ranged from 1.27±0.35% (F1) to 4.16±1.32% (F9). The moisture absorption was found to be higher in formulations F7, F8 and F9; which might be due to higher HPMC content. The lower moisture content in the formulations helps them to remain stable and becoming a completely dried and brittle film. Again, low moisture uptake protects the material from microbial contamination and bulkiness (25).

#### WVTR Study

The results of WVTR of patches are shown in table 2. The WVTR ranged from 3.42 x 10^−3^ to 6.81 x 10^−3^ gm/cm^2^. Formulation F9 showed maximum water permeation, which might be due to higher content of HPMC and allowed more WVT through the patches than other patches. The order of WVT were F9>F4>F3>F2>F5>F8>F7>F1> F6.

#### Mechanical Properties

The tensile testing shows the film's strength and elasticity, as it was evident by the parameters of tensile strength (TS), elastic modulus (EM), and elongation at break (E/B). A soft and weak polymer is characterized by a low TS, EM, and E/B; a hard and brittle polymer is defined by a moderate TS, high EM and low E/B; a soft and tough polymer is characterized by a moderate TS, low EM and high E/B; whereas a hard and tough polymer is characterized by a high TS, EM, and E/B (26). Another parameter which has been used as an indicator of the film's overall mechanical quality is strain (27). A high strain value indicates that the film is strong and elastic. Hence, it is suggested that a suitable transdermal film should have a relatively high TS, E/B, and strain but a low EM. The results of mechanical properties (TS, E/B, EM and strain) are shown in table 3. The optimized formulation F6 exhibited TS and EM values (4.34±0.16 kg/mm^2^ and 10.74±0.46 kg/mm^2^) respectively, which were significantly (P<0.05) different from those of other formulations. These results revealed that as the concentration of HPMC increased, the TS and EM also increased but E/B values decreased. An inverse relation was observed between TS and E/B. These observations indicate that formulation F6 was strong, but not brittle, and flexible.

**Table 3:** Tensile strength, Elongation at break, Elastic modulus, Strain of DOM transdermal patches

Formulation	Tensile strength (Kg/mm^2^)	Elongation at break (% mm^−2^)	Elastic modulus (Kg/mm^2^)	Strain
F1	2.04±0.12	7.89±2.10	5.25±1.54	0.38±0.04
F2	2.12±0.13	9.58±2.42	6.42±1.94	0.33±0.03
F3	2.23±0.11	8.86±2.34	6.59±1.44	0.34±0.05
F4	2.27±0.15	11.5±1.98	8.75±1.36	0.26±0.08
F5	2.90±0.13	12.5±1.56	9.44±0.98	0.30±0.06
F6	4.34±0.16	14.6±1.82	10.7±0.46	0.40±0.04
F7	4.50±0.09	15.2±1.57	11.2±0.27	0.40±0.05
F8	4.65±0.11	16.3±1.84	11.2±0.14	0.41±0.09
F9	4.85±0.12	17.8±1.93	12.3±0.23	0.39±0.08

Values represent Mean±SD (n=3).

#### In vitro release studies

**Figure 3:**
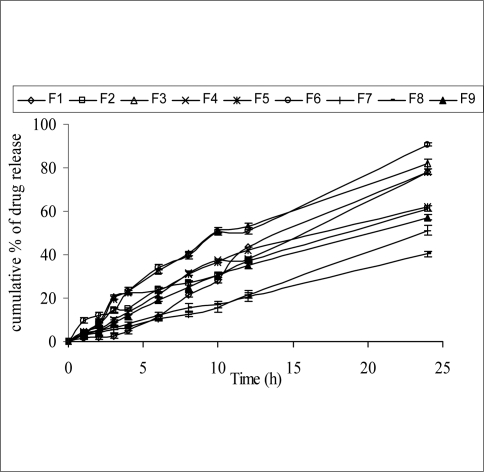
Cumulative percentage of drug release profiles from DOM transdermal patches.

The drug release profiles of DOM from transdermal patches are represented in figure 3 and table 4. From the results and plots it is clear that the drug release was governed by polymer content and permeation enhancer content. An increase in the polymer content was associated with decrease in drug release rates. Formulation F6 exhibited maximum (90.7%), and formulation F8 showed the minimum percent of drug release (40.2%) in24 hrs. The order of drug release was found to be F8<F7<F9<F5<F4<F2<F1< F3< F6. The description of drug release profiles by a model function has been attempted using zero order and first order release pattern using Korsmeyer equation (7).

7 $${\text{M}}_{\text{t}}/\text{M}\alpha =\text{K.}{\text{t}}^{\text{n}}$$

**Table 4:** In vitro Release, Ex vivo Skin Permeation, Flux, Permeability Coefficient and Lag time of DOM bilayered transdermal patches.

Code	Q_24_^a^ Release (mg)	Q_24_^b^ Permeation (μ.g cm^−2^)	Flux^c^ (μg cm^−2^h^−1^)	Kp^d^ (cm h^−1^ × 10^−2^)	LT^e^ (h)
F1	7.82±1.11	4785.6±12.5	64.5±0.27	0.64±0.007	0.25±0.005
F2	6.11±1.10	3758.5±41.2	45.8±0.24	0.45±0.015	1.20±0.014
F3	8.18±2.35	4399.6±40.6	54.2±1.32	0.54±0.027	1.14±0.241
F4	7.82±1.05	3650.2±38.7	44.6±0.28	0.44±0.046	0.85±0.093
F5	6.18±0.85	5846.2±23.1	70.4±1.87	0.70±0.015	0.69±0.068
F6	9.07±0.90	6806.6±51.2	86.2±1.85	0.86±0.056	0.94±0.032
F7	5.11±2.15	2713.0±52.5	32.6±2.13	0.32±0.039	1.70±0.007
F8	4.02±1.18	4018.1±43.2	49.3±0.19	0.49±0.005	0.26±0.104
F9	5.71±1.34	2978.1±39.5	36.9±0.5	0.36±0.041	1.50±0.216

a Cumulative amount of drug released; Mean±SD (n=3).

b Cumulative amount (µg) of drug permeated; Mean±SD (n=3).

c Flux; Mean±SD (n=3).

d Kp, Permeability coefficient; Mean±SD (n=3).

e LT, Lag time; Mean±SD (n=3).

Where Mt/Mα is the fractional release of drug, Mt is the amount which is released at time t, Mα is the total amount of drug which was present in the patches, t is the release time, K is the kinetic constant and n is the release exponent indicative of the operating release mechanism. The in vitro release data of all formulations fitted well into the Zero order equation, correlation coefficient values were between 0.925 and 0.996. The release pattern was found to follow anomalous transport mechanism, as it was evident from the release exponent (n) which was found to be in the range of 0.32 to 0.73.

#### Ex vivo permeation studies

**Figure 4:**
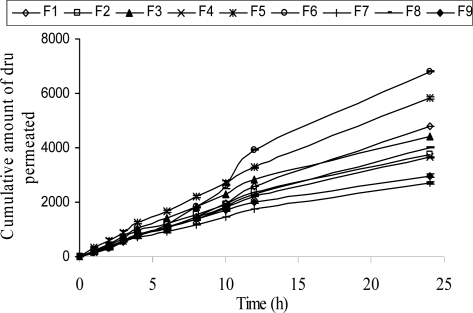
Cumulative amount of domperidone permeated from transdermal patches through rat abdominal skin.

The results of ex vivo skin permeation of DOM from patches are shown in figure 4 and table 4. The formulation F6 exhibited maximum amount (6806.64 µg) of drug permeated in 24 hrs with a flux of 86.02 µg/ h/cm (with a permeation coefficient of 0.86×10^−2^ cm/h). Plotting the cumulative amounts of drug permeated per square centimeter of the patches through the rat abdominal skin against time showed that, the permeation profiles of drug might follow zero order kinetics as it was evident by correlation coefficients 0.960 to 0.996, better fit than first order (r^2^=0.603 to 0.775) and Higuchi model (r^2^=0.865 to 0.958). The required flux for DOM was approximately 88.28 µg/cm2/hr and was obtained by formulation F6 (86.02±1.85 µg/cm^2^/hr). In order to reach the required flux, the patch area has to be increased upto 3.79 cm^2^. The results of drug permeation from transdermal patches of DOM through the rat abdominal skin confirmed that DOM was released from the formulation and permeated through the rat skin and hence could possibly permeate through the human skin.

#### Drug-polymer interaction study

**Figure 5:**
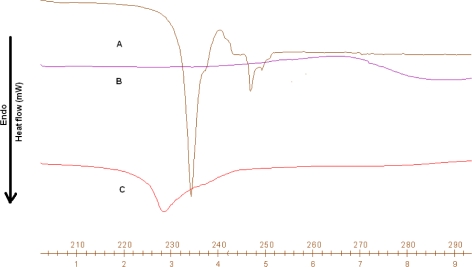
DSC thermograms of DOM (a), HPMC E15 (b), Physical mixture (c).

**Figure 6:**
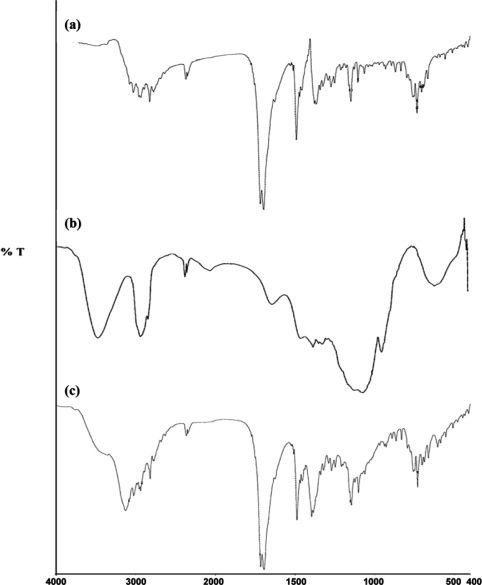
FTIR Spectra of DOM (a), HPMC E15 (b), Physical mixture (c).

DSC analysis of domperidone, HPMC, and physical mixture are shown in figure 5. DOM exhibited a sharp endothermic event as a melting peak with the onset temperature of 234.6 °C (△H=138.9 J/g). The thermal behavior of HPMC exhibited no such phenomenon in any temperature intervals. The appearance of a peak corresponding to the melting of DOM was also evident in the thermogram of the physical mixture. The results revealed a negligible change in the melting point of domperidone in the presence of polymeric materials. FTIR spectra of DOM (Figure 6) exhibited principal peaks at 3025.06 cm-1 (N-H stretching), 2818.07cm^−1^ (asymmetric C–H stretching), 1715.31 cm^−1^, 1694.22 cm^−1^ (C=O stretching), and 1489.15 cm^−1^ (N=C stretching peak). There were some other characteristic peaks which were observed at 1489.15 cm^−1^, 1147.18 cm^−1^and 1062.18 cm^−1^. The FTIR spectrum of HPMC presented a profile without distinctly high peaks. The physical mixture showed approximate superimposition of the drug and HPMC. Both the DSC and FTIR results suggest that there was no interaction between drug and polymer which were used in the present study.

#### Stability study

The stability of the optimized formulation (F6) was investigated as per ICH guidelines. The formulation was stored at a temperature 40±0.5 oC and 75±5% RH for 6 months. There was no significant change in release and drug content. Only a 3.19% of degradation (lesser than initial drug content) was observed. As the degradation of the formulation is less than 5%, a shelf life of 2 years may be expected.

## CONCLUSIONS

Based on the results of the present study, it may be concluded that selected polymers were better suited for development of bilayered matrix type transdermal patches of DOM. The formulation F6 showed maximum release and flux (86.02 µg/cm^2^/hr) which is closely related to the target flux (88.28 µg/cm^2^/hr). In order to reach the target flux, the patch area has to be increased to 3.79 cm^2^. Further work is recommended to support its efficacy claims by long term pharmacokinetic and pharmacodynamic studies on human beings.
